# Protective Effects of a Polyphenolic Fraction of Bergamot in Improving the Welfare Index in Honeybees (*Apis mellifera*) Co-Exposed to Deltamethrin and Flupyradifurone: A Laboratory Cage Study

**DOI:** 10.3390/ani16152374

**Published:** 2026-08-03

**Authors:** Roberto Bava, Fabio Castagna, Rosa Maria Bulotta, Stefano Ruga, Giovanna Liguori, Carmine Lupia, Saverio Nucera, Carolina Muscoli, Vincenzo Mollace, Ernesto Palma

**Affiliations:** 1 Department of Health Sciences, University “Magna Graecia” of Catanzaro, 88100 Catanzaro, Italy; roberto.bava@unicz.it (R.B.); fabiocastagna@unicz.it (F.C.); rmbulotta@unicz.it (R.M.B.); palma@unicz.it (E.P.); 2 Local Health Authority (ASL) Foggia, 71121 Foggia, Italy; giovanna.liguori@aslfg.it; 3 Sbarro Institute for Cancer Research and Molecular Medicine, Center for Biotechnology, Department of Biology, College of Science and Technology, Temple University, Philadelphia, PA 19122, USA; 4 Mediterranean Ethnobotanical Conservatory, 88054 Sersale, Italy; studiolupiacarmine@libero.it; 5 Center for Pharmacological Research, Food Safety, High Tech and Health (IRC-FSH), University “Magna Graecia” of Catanzaro, 88100 Catanzaro, Italy; saverio.nucera@unicz.it (S.N.); muscoli@unicz.it (C.M.); mollace@unicz.it (V.M.)

**Keywords:** pesticide interaction, antagonism, oxidative stress, detoxification, bergamot flavonoids, nutritional supplementation, pollinator conservation, cage trial

## Abstract

The simultaneous presence of multiple pesticide residues in the environment impairs honeybee health and survival. The toxicological interactions could result in additive, synergistic, or antagonistic effects as documented in previous studies. In most cases, the co-exposure produces an amplification of the singular toxicity, resulting in additive or synergistic effects. More rarely, the interaction of active principles leads to antagonistic effects. Previous studies demonstrated that deltamethrin (DMT, a type II pyrethroid) and flupyradifurone (FLU, a butenolide insecticide) produce antagonistic toxicity when co-administered orally to caged *Apis mellifera* workers, and that dietary supplementation with the bergamot polyphenolic fraction (BPF) independently protects against FLU- and DMT-induced intoxication. The present study investigates the three-way interaction of DMT, FLU, and BPF on honeybees. Survival rate, solution consumption, and abnormal behavior have been recorded over a 72-h exposure period in groups of twenty caged honeybees orally treated with DMT (21.6 mg/L), FLU (50 or 100 mg/L), their binary combinations, or their ternary combinations with BPF (1 mg/kg). Results demonstrate that BPF alone did not differ from control (*p* > 0.05) at any time point, confirming its safety. Binary combinations of DMT and FLU showed antagonistic survival profiles consistent with our previous report. The addition of BPF to both binary pesticide combinations significantly improved survival (*p* < 0.001), reduced abnormal behavioral frequencies (*p* < 0.05), and increased solution intake (*p* < 0.05) compared to the respective DMT + FLU groups. These results suggest that BPF exerts a protective effect that extends to complex multi-pesticide exposure scenarios. These effects may be associated with antioxidant and detoxification-related pathways, although further biochemical and molecular analyses are required to elucidate the underlying mechanisms.

## Simple Summary

In the environment, honeybees are often exposed to several categories of pesticides. This co-exposure could produce different results. In recent research, the interaction between two widely used agrochemicals, deltamethrin (DMT) and flupyradifurone (FLU), was previously shown to produce positive effects on bee survival, determining an improvement of this index. Otherwise, the integration of diet with supplements rich in polyphenols is well known to reduce intoxication phenomena induced by pesticide exposure. Building on this evidence, the present study investigates whether dietary supplementation with the bergamot polyphenolic fraction (BPF) can further attenuate the combined toxicity of the abovementioned pesticides. Our results demonstrate that BPF significantly improved survival rates and reduced abnormal behaviours in bees co-exposed to deltamethrin and flupyradifurone under laboratory conditions. These findings highlight the potential of BPF-enriched dietary supplements as a vehicle for supporting honeybee health in agricultural environments.

## 1. Introduction

Honeybee populations are slowly declining, not attributable to a single factor, but to multiple interacting agents that amplify each other’s harmful effects [1,2]. Among the main predisposing factors are climate change, damage caused by various pathogens, and the impoverishment of terrestrial habitats due to intensive agriculture and the spread of monocultures [3,4]. These practices reduce the availability of nutritional resources [5]. Furthermore, widespread monocultures also lead to increased competition among insects and consequent selection, with some species becoming predominant over others [6].

In these artificially altered environments, pesticides are increasingly used: to protect crops from harmful species, farmers have increasingly resorted to their use [7]. Bees, among the most efficient pollinators, are used for pollination both in greenhouses and in open fields [8]. Their crucial role in ensuring good harvests, both in terms of quantity and quality, makes them particularly vulnerable to chemicals used in agriculture [2,8]. Often, they are not only exposed to a single toxic substance but must also endure the damage caused by co-exposure to complex mixtures of agrochemicals [9].

The toxicological consequences of such exposures to multiple pesticides have been little investigated and are therefore still poorly understood [10]. Toxicological interactions between pesticides present at the same time can profoundly modify their individual toxicity profiles, giving rise to additive, synergistic, or, on the contrary, antagonistic effects [11]. Indeed, numerous studies demonstrate that co-exposure to fungicides and insecticides aggravates toxicity compared to single exposures, causing acute, subacute, and chronic damage [12,13].

Oral exposure to sublethal concentrations of the tebuconazole + fluopyram mixture, for example, dose-dependently reduces survival, body weight, and food consumption (syrup and pollen) in female workers, while altering antioxidant enzymes and inducing oxidative stress [14]. Alongside these additive and synergistic effects, well documented in the literature, antagonistic interactions also occur, in which co-exposure results in lower toxicity than single exposure [15]. In these latter cases, the toxic effect resulting from co-exposure is less severe than that resulting from single exposure to the toxicant.

A notable case of antagonism was recently reported by Bava et al. [16], who studied the effects of simultaneous exposure to sublethal doses of flupyradifurone and deltamethrin on caged bees. The researchers found that experimental groups in which the bees were exposed to the toxic mixture showed improved survival rates and a reduction in abnormal behavior over a 72-h exposure period compared to groups treated with deltamethrin and flupyradifurone alone. This was likely due to a form of functional antagonism, where DMT-induced delayed inactivation of voltage-gated sodium channels leading to persistent neuronal excitation is counteracted by FLU-induced sustained activation of nicotinic acetylcholine receptors (nAChRs), resulting in a compensatory desensitization of overstimulated neurons and the activation of compensatory metabolic detoxification pathways [16]. Regarding the ecological relevance of the selected pesticide concentrations, it is important to clarify their toxicological rationale. DMT at 21.6 mg/L (equivalent to approximately 43.2 µg/bee assuming a daily intake of approximately 2 µL) represents an intentionally toxic benchmark dose selected to produce complete mortality within 72 h, thereby allowing the detection of antagonistic or protective effects. While this concentration exceeds typical field-relevant residues—reported in nectar and pollen at low ng/g to sub-µg/g levels [9]—it falls within the range of worst-case exposure scenarios such as direct spray contamination or consumption of guttation fluid from treated crops, where concentrations up to several mg/L have been documented [17,18]. FLU at 50 mg/L approaches the upper limit of sublethal exposure, while FLU at 100 mg/L approximates the acute oral LD_50_ for caged honeybees under standardized testing conditions [19]. Thus, the selected doses span a continuum from sublethal to acutely toxic, enabling the assessment of both antagonistic pesticide-pesticide interactions and BPF-mediated protection across a range of toxicological pressures. In other previous and independent studies, Bava et al. investigated the ability of bergamot polyphenolic fraction (BPF), a standardized, polyphenol-rich extract derived from the juice and pith of Citrus bergamia Risso et Poiteau, to reduce DMT- [20] and FLU- induced [21] lethality and behavioral toxicity in caged bees. These findings, along with broader evidence that dietary polyphenols and flavonoids can upregulate detoxification enzymes and improve physiological status in honeybees under nutritional and xenobiotic stress [22,23,24], provide the rationale for this study. In the present work, we adopt a composite “welfare index” approach, integrating survival, solution consumption, and behavioral parameters as complementary indicators of overall bee health.

Indeed, the present paper represents a natural continuation of this line of research and aims to investigate the three-way interaction between DMT, FLU, and BPF in caged bees under controlled laboratory conditions. Specifically, we evaluated whether dietary supplementation with BPF to binary DMT + FLU combinations, already characterized as antagonistic, could further improve bee survival, reduce the incidence of abnormal behaviors, and restore solution intake over a 72-h observation period. This experimental model provides a more ecologically realistic representation of the multiple stress scenarios honey bees encounter in agricultural environments and may contribute to the development of evidence-based feeding strategies to support hive health.

## 2. Materials and Methods

### 2.1. Honeybee Collection and Housing

This study was conducted at the Department of Health Sciences, University “Magna Graecia” of Catanzaro, Italy, during the summer of 2025, in accordance with established guidelines for the avoidance of physiologically altered bees [25]. Experimental honeybees were collected from three healthy colonies of *Apis mellifera* managed with standard beekeeping procedures and subjected to prior health inspection. Brood combs containing emerging honeybees were placed in an incubator at 35 °C and 65–80% relative humidity; newly emerged workers were collected after 12 h to ensure age homogeneity [25]. Bees were gently brushed, mixed, and divided into groups of 20 individuals housed in standard cages equipped with horizontal feeders (sterile 2.5 mL disposable syringes with capped ends) [26]. Cages were maintained at 33 ± 2 °C and 70% relative humidity in the dark. Before treatment, bees were allowed a 24-h acclimation period with ad libitum access to 50% (*w*/*v*) sucrose solution [26].

### 2.2. Experimental Groups and Treatment Administration

The following nine experimental groups were established:

(1) Control group (CTRL), receiving sucrose solution only;
(2) Bergamot polyphenolic fraction group (BPF), fed with BPF at 1 mg/kg of feed (syrup) in sucrose solution;
(3) Deltamethrin group (DMT 21.6), intoxicated with deltamethrin at 21.6 mg/L in sucrose solution;
(4) Flupyradifurone group (FLU 50), intoxicated with flupyradifurone at a dose of 50 mg/L in sucrose solution;
(5) Flupyradifurone group (FLU 100), intoxicated with flupyradifurone at 100 mg/L in sucrose solution;
(6) A group receiving a binary combination of deltamethrin and a low dose of flupyradifurone (DMT 21.6 + FLU 50);
(7) A group receiving a binary combination of deltamethrin and a high dose of flupyradifurone (DMT 21.6 + FLU 100);
(8) A group receiving a ternary combination of deltamethrin, low dose of flupyradifurone, and BPF (DMT 21.6 + FLU 50 + BPF);
(9) A group receiving a ternary combination of deltamethrin, high dose of flupyradifurone, and BPF (DMT 21.6 + FLU 100 + BPF).

Ten replicates (cages) per experimental group were prepared, each containing 20 bees (200 bees per group).

Pesticide concentrations were selected based on previous studies [20,21,22]. DMT (21.6 mg/L) represents an acutely toxic dose causing complete mortality within 72 h [21]. FLU at 50 mg/L is sublethal, while FLU at 100 mg/L approaches the LC50 [22]. BPF was administered at 1 mg/kg, the dose previously found to be non-toxic and protective [21,22]. All stock solutions were prepared in deionized water, stored at 2–6 °C protected from light, and renewed every three days. Feeders were replaced every 24 h. Treatment solutions were administered orally via feeders.

### 2.3. Preparation of BPF

BPF was produced from the fruits of *Citrus bergamia* Risso et Poiteau collected in the Bianco-Reggio Calabria area (Calabria, Italy). Juice was extracted from peeled fruits by squeezing, then processed through oil fraction depletion by stripping, clarification by ultra-filtration, and loading onto a polystyrene resin column selective for polyphenols with molecular weights between 300 and 600 Da. Polyphenol fractions were eluted with KOH solution; the eluate was incubated with agitation to reduce furocoumarin content, then neutralized by filtration through cationic resin at acidic pH. The resulting phytocomplex was vacuum-dried and sized to pass a 70 mesh, yielding BPF powder [27].

### 2.4. BPF Analysis

The BPF was characterized by HPLC/HRMS (Orbitrap Q-Exactive). The UHPLC/HRMS analysis was performed on a Dionex Ultimate 3000 RS system coupled to a Thermo Scientific Q-Exactive Orbitrap mass spectrometer (Thermo Fisher Scientific, Brema, Germany). Chromatographic separation was achieved on a Thermo Scientific Hypersil Gold C18 column (150 × 2.1 mm, 1.9 µm particle size) equilibrated with 95% solvent A (0.1% formic acid in water) and 5% solvent B (methanol). The elution flow rate was 200 µL/min with a linear gradient from 5% to 100% solvent B over 45 min, followed by an isocratic hold at 100% for 2 min, and re-equilibration at 5% for 13 min (total run time 60 min).

The autosampler and column oven were maintained at 20 °C and 24 °C, respectively. Electrospray ionization was performed in negative polarity mode with the following source parameters: spray voltage 2.9 kV, sheath gas 30 arbitrary units, auxiliary gas 10, probe heater temperature 280 °C, capillary temperature 320 °C, and S-lens RF level 50. Full scan mass spectra were acquired at 35,000 resolving power (FWHM at m/z 200) over the mass range 550–850 amu. Data-dependent MS^2^ (ddMS^2^) acquisition was performed at 17,500 resolving power with an isolation window of 0.4 m/z and normalized collision energy of 25.

### 2.5. Assessment of Survival, Consumption, and Abnormal Behavior

Survival and solution consumption were recorded every 24 h over 72 h. Consumption per bee was calculated by dividing the total volume consumed over each 24-h interval by the number of bees alive at the end of that interval. This approach provides a conservative estimate of individual intake and is consistent with standard cage-trial protocols, although it does not account for the exact timing of mortality within the interval. Evaporation effects were equally distributed across all groups and thus not corrected for, in accordance with standard protocols. Abnormal behaviors were assessed at 1, 2, 4, 24, 48, and 72 h post-treatment by a single trained observer who was blinded to treatment allocation. The observer scored each bee individually for 6 s per cage (up to 120 s per cage of 20 bees). Behaviors were classified according to OECD guidelines [19] into the following categories: motor incoordination, curved-down abdomen, hyperactivity, reduced locomotor activity, and moribundity. The number of bees displaying each category of abnormal behavior was recorded, and the total proportion of bees exhibiting any abnormal behavior per cage was used as the primary behavioral endpoint. Dead bees were excluded from the denominator at each observation time point, whereas moribund bees were included and scored as abnormal. All behavioral assessments were conducted by the same observer throughout the study, ensuring internal consistency; inter-observer reliability was therefore not applicable to this single-observer design. For the purposes of this study, the term “welfare index” refers to the integrated assessment of these three complementary endpoints—survival, consumption, and behavior—evaluated concurrently. No formal aggregated score was calculated; rather, each endpoint was analyzed independently and interpreted collectively to provide a holistic picture of bee health status [19].

### 2.6. Statistical Analysis

Survival data were analyzed using Kaplan–Meier curves and compared with the log-rank test. For all analyses, the cage (n = 10 per treatment group) was treated as the experimental unit. Survival was recorded at the individual bee level within each cage, and Kaplan–Meier analysis was performed on pooled individual data with censoring for bees surviving at the end of the observation period. Solution consumption and abnormal behavior frequencies were calculated per cage, and statistical comparisons were performed using cage-level values. For pairwise comparisons between experimental groups, Bonferroni correction was applied to account for multiple testing (adjusted α = 0.05/11 ≈ 0.0045, based on the 11 pre-planned comparisons of interest). Data distribution was assessed using the Shapiro–Wilk normality test. Since data deviated significantly from normality (*p* < 0.05), differences in solution consumption and abnormal behavior frequencies were evaluated using the Kruskal–Wallis test followed by Dunn’s post hoc test with Bonferroni correction for multiple comparisons; for these analyses, adjusted *p*-values < 0.05 were considered statistically significant. All statistical analyses were performed using GraphPad Prism (version 10; GraphPad Software Inc., La Jolla, CA, USA). Results are expressed as mean ± SEM, and statistical significance was set at *p* < 0.05 (after adjustment where applicable).

## 3. Results

### 3.1. BPF Composition and Quality Control

The three main flavonoids detected were neoeriocitrin (370 ppm), naringin (520 ppm), and neohesperidin (310 ppm), which together constitute the majority of the detected flavonoid content, representing approximately 3.16% of the total BPF powder weight. Total polyphenol content was 38%. Microbiological quality of the BPF powder was assessed according to the following ISO standards: ISO 4833-1:2013 (aerobic plate count) [28], ISO 21527-1:2008 (yeasts and moulds count) [29], ISO 16649-2:2001 (*Escherichia coli*) [30], ISO 4832:2006 (coliforms) [31], UNI EN ISO 6579:2000 (*Salmonella* spp.) [32], and UNI EN ISO 6888-2:2004 (*Staphylococcus aureus*) [33]. Aerobic plate count was <1000 CFU/g; yeasts and moulds count was <100 CFU/g; *Escherichia coli*, *coliforms*, *Salmonella* spp., and *Staphylococcus aureus* were absent in 25 g of sample. Mycotoxin analysis (aflatoxins B1, B2, G1, G2, and ochratoxin A) was performed according to UNI EN ISO 16050:2011 [34] and showed levels below the limit of detection (LOD < 0.5 µg/kg for aflatoxins, <1.0 µg/kg for ochratoxin A). Pesticide residues (panel of 500+ molecules) and heavy metals (lead, cadmium, mercury, arsenic) were analyzed by LC-MS/MS and ICP-MS, respectively, and were below the respective regulatory thresholds established by the European Pharmacopoeia for food-grade botanical extracts.

### 3.2. Effects on Honeybee Survival

Over the 3-day experimental period, CTRL honeybees showed a stable survival rate, with 92.4% survival at the end of the 72-h experimental period. Similarly, the BPF-treated group did not differ significantly from CTRL (*p* > 0.05). In contrast, honeybees exposed to DMT 21.6 exhibited significantly reduced survival from day 1 compared to CTRL (*p* < 0.001). A significant reduction was also observed in the FLU 50 group (*p* < 0.001 vs. CTRL). The FLU 100 treatment had a more pronounced effect, with only 17.3% survival at day 1 and complete mortality by day 2; accordingly, survival was significantly lower than both CTRL and FLU 50 (*p* < 0.001).

Co-exposure to DMT 21.6 and FLU 50 significantly improved survival compared to both DMT 21.6 and FLU 50 alone (*p* < 0.001). In contrast, the DMT 21.6 + FLU 100 group showed a higher survival rate than DMT 21.6 alone at day 1, but this effect was not sustained over time, resulting in 100% mortality by day 3, similar to DMT 21.6 alone (*p* > 0.05).

**Figure 1:**
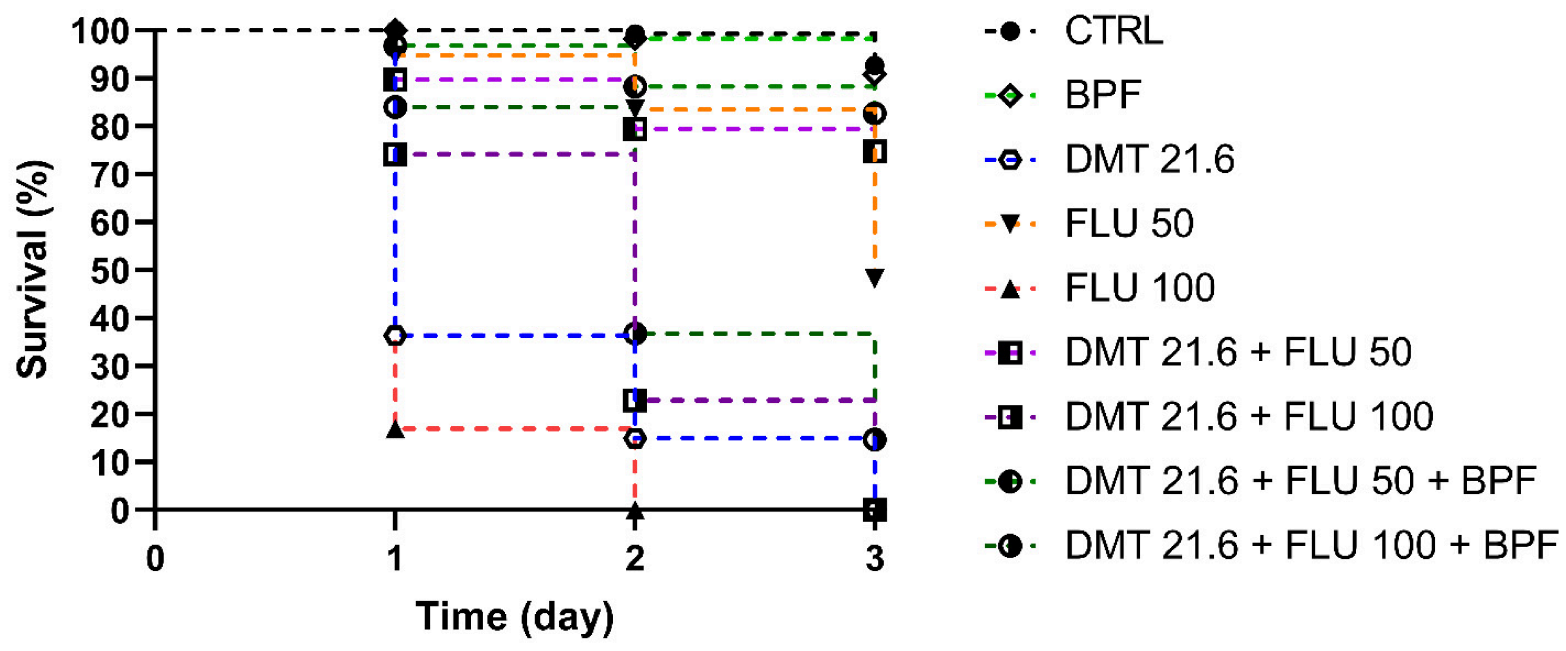
Kaplan–Meier survival curves of honeybees exposed to different treatments. CTRL: control; BPF: bergamot polyphenolic fraction; DMT: deltamethrin, FLU: flupyradifurone. BPF vs. CTRL: *p* > 0.05; DMT 21.6 vs. CTRL: *p* < 0.001; FLU 50 vs. CTRL: *p* < 0.001; FLU 100 vs. CTRL: *p* < 0.001; FLU 100 vs. FLU 50: *p* < 0.001; DMT 21.6 + FLU 50 vs. DMT 21.6: *p* < 0.001; DMT 21.6 + FLU 50 vs. FLU 50: *p* < 0.001; DMT 21.6 + FLU 100 vs. DMT 21.6: *p* > 0.05; DMT 21.6 + FLU 100 vs. FLU 50: *p* < 0.001; DMT 21.6 + FLU 50 + BPF vs. DMT 21.6 + FLU 50: *p* < 0.001; DMT 21.6 + FLU 100 + BPF vs. DMT 21.6 + FLU 100: *p* < 0.001.

Notably, the addition of BPF significantly increased survival in both combined treatments, with DMT 21.6 + FLU 50 + BPF and DMT 21.6 + FLU 100 + BPF showing higher survival rates compared to their respective combinations without BPF (*p* < 0.001) (Figure 1, Table 1).

### 3.3. Effects on Solution Consumption

**Figure 2:**
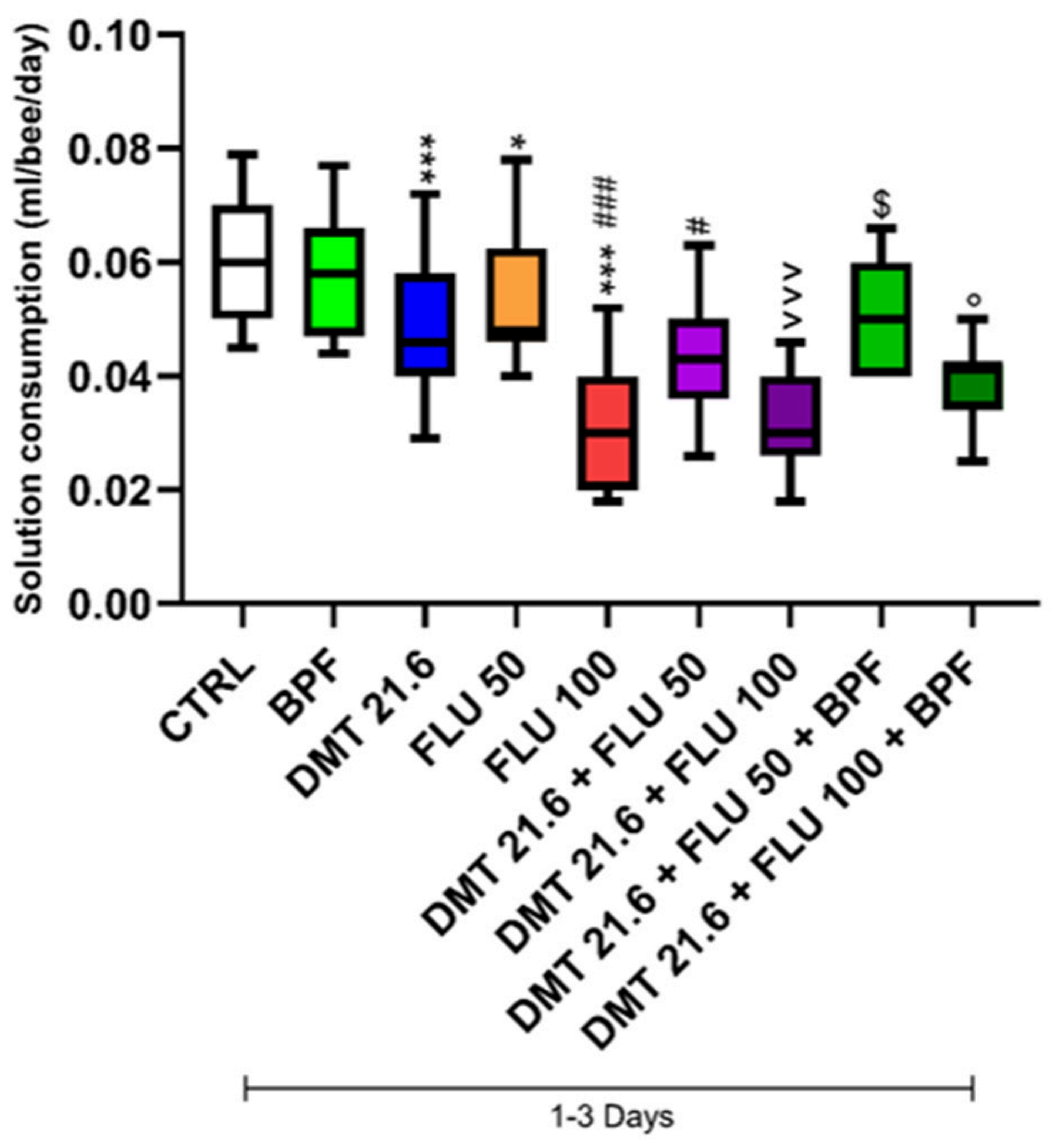
Solution consumption in honeybees over the 3-day experimental period. CTRL: control; BPF: bergamot polyphenolic fraction; DMT: deltamethrin; FLU: flupyradifurone. * *p* < 0.05 vs. CTRL; *** *p* < 0.001 vs. CTRL; # *p* < 0.05 vs. FLU 50; ### *p* < 0.001 vs. FLU 50; ^^^ *p* < 0.001 vs. DMT 21.6; $ *p* < 0.05 vs. DMT 21.6 + FLU 50; ° *p* < 0.05 vs. DMT 21.6 + FLU 100. The vertical line reports the minimum and maximum values.

Solution consumption over the experimental period (3 days) is shown in Figure 2. No significant differences were observed between the CTRL and BPF groups (*p* > 0.05). In contrast, bees exposed to DMT 21.6 showed a significant reduction in solution intake compared to CTRL (*p* < 0.001). A similar decrease was observed in the FLU 50 group (*p* < 0.05 vs. CTRL), while FLU 100 further reduced consumption, with values significantly lower than both CTRL (*p* < 0.001) and FLU 50 (*p* < 0.001).

Co-exposure to DMT 21.6 and FLU 50 resulted in a significant decrease in intake compared to FLU 50 alone (*p* < 0.05), and the combination of DMT 21.6 and FLU 100 similarly reduced consumption relative to FLU 100 (*p* < 0.001). Notably, the addition of BPF to the combined treatments significantly increased solution consumption compared to the respective DMT + FLU groups without BPF (*p* < 0.05 for both comparisons).

### 3.4. Effects on Abnormal Behavior

**Figure 3:**
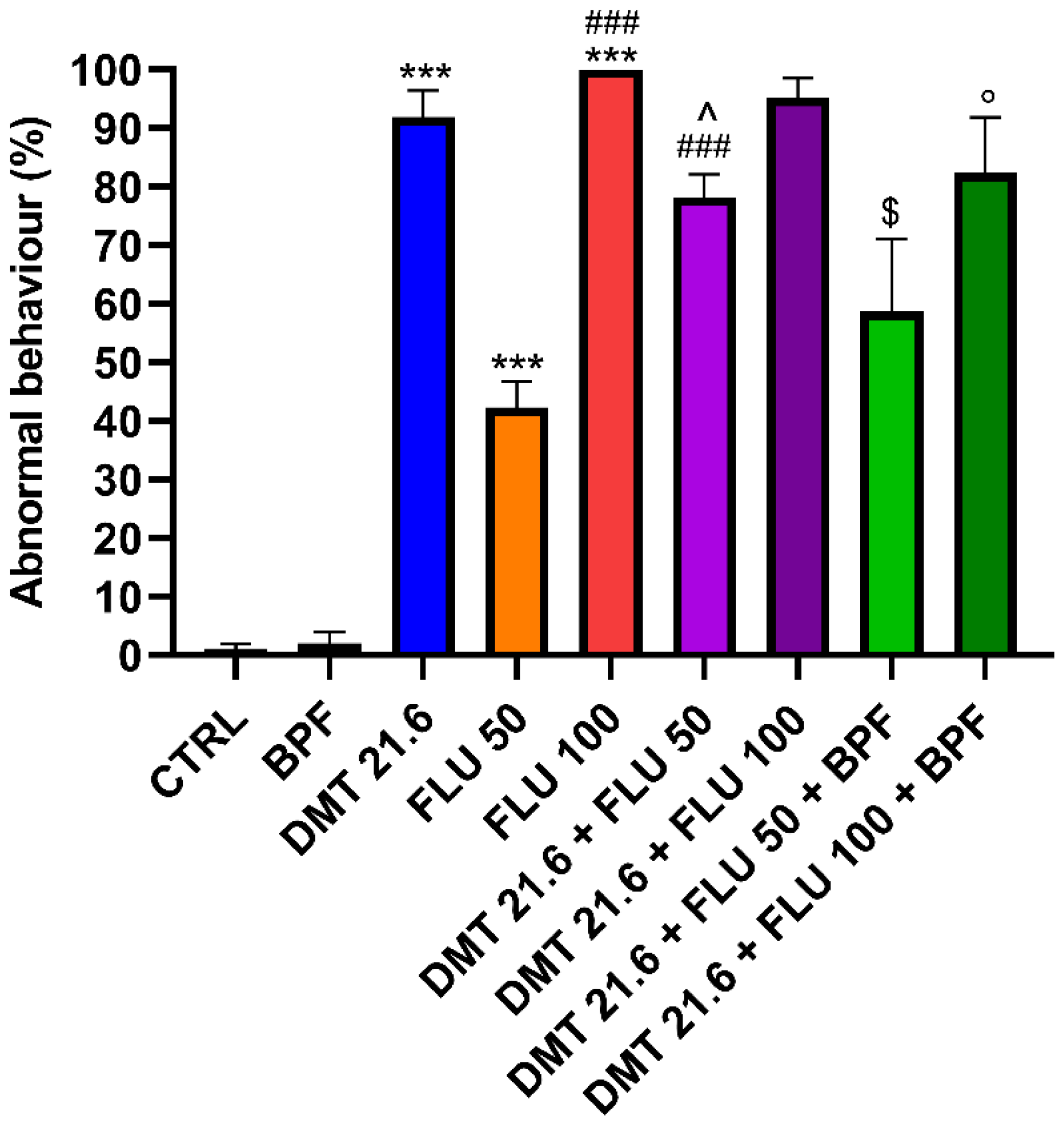
Abnormal behavior in honeybees following exposure to different treatments. CTRL: control; BPF: bergamot polyphenolic fraction; DMT: deltamethrin, FLU: flupyradifurone. *** *p* < 0.001 vs. CTRL; ### *p* < 0.001 vs. FLU 50; ^ *p* < 0.05 vs. DMT 21.6; $ *p* < 0.05 vs. DMT 21.6 + FLU 50; ° *p* < 0.05 vs. DMT 21.6 + FLU 100.

Abnormal behavior over the experimental period (3 days) is shown in Figure 3. No significant differences were observed between CTRL and BPF groups (*p* > 0.05). In contrast, bees exposed to DMT 21.6 showed a significant increase in abnormal behaviors compared to CTRL (*p* < 0.001). A similar increase was observed in the FLU 50 group (*p* < 0.001 vs. CTRL), while FLU 100 further enhanced abnormal behaviors, with values significantly higher than both CTRL (*p* < 0.001) and FLU 50 (*p* < 0.001).

Co-exposure to DMT 21.6 and FLU 50 resulted in a significant reduction in abnormal behaviors compared to both FLU 50 alone (*p* < 0.001) and DMT 21.6 alone (*p* < 0.05), whereas the combination of DMT 21.6 and FLU 100 did not reduce the elevated levels observed with FLU 100. Notably, the addition of BPF significantly decreased abnormal behaviors in both combined treatments compared to the respective DMT + FLU groups without BPF (*p* < 0.05 for both comparisons).

Qualitatively, the pattern of abnormal behaviors varied across treatment groups. In bees exposed to DMT 21.6 alone, hyperactivity and motor incoordination were the most frequently observed abnormal behaviors in the initial phase, progressively evolving toward moribundity by 48–72 h. Conversely, FLU 100 induced a rapid onset of apathy and reduced locomotor activity, with most bees becoming moribund within 24 h. The binary combination DMT 21.6 + FLU 50 markedly reduced the frequency of all categories of abnormal behavior compared to the single exposures. Notably, BPF supplementation in both ternary combinations further decreased the proportion of bees displaying severe motor impairment and moribundity, with a corresponding increase in bees exhibiting normal locomotor activity and grooming behavior.

## 4. Discussion

The honeybee die-off observed in recent decades is a multifactorial phenomenon driven by parasites, pathogens, nutritional deficits, and pesticide exposure [35]. Among these, the simultaneous exposure to multiple pesticides is of particular concern, as it creates unpredictable toxicological interactions that single-compound risk assessments fail to capture [36,37]. In this context, our previous work demonstrated that DMT and FLU, two distinct but neurologically convergent insecticides, produce antagonistic effects when co-administered to caged honeybees [16]. DMT, as a pyrethroid, acts by delaying the closure of voltage-gated sodium channels, causing persistent membrane depolarization. FLU, as an AChR agonist, competes with acetylcholine at the synaptic receptor, generating a sustained cholinergic signal. We hypothesized that the concurrent overstimulation of two distinct neuronal targets leads to a compensatory desensitization that paradoxically results in reduced overall toxicity, a phenomenon that may also involve the hormesis principle at higher doses [16].

The present data confirm and extend this antagonistic pattern. The DMT 21.6 + FLU 50 binary combination produced substantially higher survival rates than either compound alone (75.2% at day 3 vs. 0% for DMT alone and 49.7% for FLU 50 alone) and was associated with reduced abnormal behaviors compared to both single exposures. The DMT 21.6 + FLU 100 combination showed only a transient survival benefit on day 1, with complete mortality by day 3, consistent with the hormesis concept: at the highest dose of FLU, the detoxification and metabolic buffering systems are overwhelmed and can no longer sustain the protective antagonistic response.

The most important new finding of this study is the significant and consistent protective effect of BPF when added to both binary pesticide combinations. BPF supplementation at 1 mg/kg was previously shown to be safe and protective against individual DMT [20] and FLU [21] toxicity in caged honeybees. Here, we demonstrate that this protection extends to multi-pesticide exposures and that BPF does not simply add to the antagonistic effect already produced by the pesticide interaction but provides a substantial additional layer of protection. In the DMT + FLU 50 + BPF group, survival at day 3 reached 82.1%, exceeding that of the DMT + FLU 50 group (75.2%) and approaching control levels. In the DMT + FLU 100 + BPF group, 13.6% of bees survived to day 3, compared to complete mortality in the group without BPF.

The mechanisms underlying BPF’s protective activity are likely multiple. Flavanone glycosides present in BPF, particularly naringin, neohesperidin, and neoeriocitrin, are well-established antioxidants capable of scavenging reactive oxygen species (ROS) and inhibiting lipid peroxidation [38]. Consistent with this mechanism, a recent preliminary study by Guo et al. (2026) [23] demonstrated that the dietary supplement Fanfengling enhances gut antioxidant capacity in *Apis mellifera* by upregulating glutathione metabolism and increasing flavonoid levels (e.g., quercetin 3-O-glucoside) under spring nutritional stress.

Although that study addressed a different stressor (forage scarcity rather than pesticide toxicity), the convergence on flavonoid-mediated antioxidant pathways supports the broader concept that plant-derived polyphenols bolster bee resilience through shared redox-regulating mechanisms [23]. Pesticide-induced toxicity is strongly associated with oxidative stress: pyrethroid exposure generates free radicals and disrupts antioxidant defense systems in both vertebrate and invertebrate models [38,39,40]. By counteracting ROS accumulation, BPF may protect neuronal membranes and mitochondria from oxidative damage, thereby preserving cellular function even in the presence of multiple neurotoxic agents [38]. Additionally, dietary polyphenols, including naringin and quercetin, have been shown to upregulate cytochrome P450 enzymes in honeybees, increasing their capacity to detoxify xenobiotics [24]. A similar mechanism may operate with BPF, allowing bees to more efficiently metabolize and eliminate DMT and FLU residues. Because oxidative stress biomarkers, detoxification enzyme activity, and pesticide residue levels were not measured in the present study, the proposed mechanisms remain hypothetical and warrant direct investigation in future work. This hypothesis is indirectly supported by the observed increase in solution consumption in BPF-supplemented groups, a proxy of general vitality and feeding motivation, which was restored toward control levels.

The behavioral data provide complementary support. BPF supplementation significantly reduced the proportion of bees displaying abnormal behaviors (motor incoordination, curved abdomen, hyperactivity, apathy, moribundity) in both BPF-containing ternary groups. This is consistent with a general improvement in neurological function, possibly reflecting reduced oxidative neuronal damage and more effective clearance of neuroactive pesticide residues.

An important consideration concerns the practical translational value of these findings. BPF is derived from bergamot, a plant cultivated almost exclusively in the Reggio Calabria province of southern Italy, and is already available as a standardized food-grade extract with demonstrated safety in multiple species [41,42,43]. The dose used in the present study (1 mg/kg) is pharmacologically active without causing any detectable toxicity, as confirmed by the BPF-alone group being statistically indistinguishable from controls across all endpoints. These characteristics make BPF a realistic candidate for incorporation into supplementary beekeeping feeds, a practice already in use with other natural bioactive compounds [23].

However, several limitations of the present study must be acknowledged. First, the relatively short, 72-h exposure period under highly controlled laboratory conditions, while essential for establishing causality, does not fully capture chronic, life-long exposure scenarios typical of field conditions. In the field, forager bees encounter fluctuating pesticide concentrations over extended periods, different developmental stages may exhibit differential sensitivity, and colony-level dynamics, such as queen reproduction, brood development, and social immunity, may modulate individual responses [44,45]. Second, only one concentration of BPF was tested; comprehensive dose–response relationships between BPF and the multi-pesticide combination remain to be explored in future work. Third, the mechanistic basis of BPF’s protective action, particularly the relative contributions of direct antioxidant scavenging versus enzymatic detoxification upregulation, remains to be elucidated in honeybees. Molecular and biochemical studies measuring ROS levels, antioxidant enzyme activities (e.g., superoxide dismutase, catalase, glutathione S-transferase), and P450 gene expression would be valuable to address this question. Fourth, field-scale validation studies are required before practical recommendations for beekeeping supplementation can be formulated. Fourth, the calculation of per-bee solution consumption by dividing total cage consumption by the number of surviving bees may introduce bias in groups with high mortality, as the timing of deaths within the 24-h interval could not be precisely accounted for. This limitation is inherent to group-housing experimental designs and may result in slight over- or underestimation of individual intake. Future studies employing individual feeding chambers could address this confound.

Finally, it is worth noting that the antagonistic interaction between DMT and FLU is itself a noteworthy and underexplored phenomenon. Most pesticide combination studies in bees report synergistic or additive effects [11], and antagonism is comparatively rare and less studied. From an applied perspective, antagonistic interactions could be exploited in the rational design of pesticide mixtures that reduce the overall environmental load of active substances while maintaining pest control efficacy. Conversely, from a risk assessment standpoint, antagonism means that evaluating binary combinations based solely on their individual toxicity data may not predict the actual biological outcome, underscoring the need for multi-compound interaction studies to become a standard component of regulatory ecotoxicology. In summary, this study extends the research line developed over the past two years on the toxicological interactions between pesticides in honeybees and on the potential of the bergamot polyphenolic fraction to mitigate pesticide-induced toxicity [16,20,21]. The present findings demonstrate, for the first time, that BPF is capable of significantly improving honeybee survival and reducing behavioral toxicity even in a complex multi-pesticide exposure scenario combining DMT and FLU.

## 5. Conclusions

The present study demonstrates that the bergamot polyphenolic fraction (BPF) significantly improves bee survival and reduces behavioral toxicity in bees exposed to the binary combination of deltamethrin and flupyradifurone. These results build on previous evidence of toxicological antagonism between DMT and FLU, and on the independent protective effects of BPF against each pesticide individually, demonstrating that BPF provides significant additional protection in a multi-pesticide scenario. The safety of BPF at the tested dose and its proven antioxidant and pro-detoxifying properties in bees make it a promising candidate for incorporation into hive supplemental nutrition strategies. The therapeutic potential of BPF could therefore be considered to promote pollinator conservation in complex ecosystems where resilience plays a crucial role. Further field studies are needed before practical recommendations can be made, but the current results provide a solid experimental basis for this research direction.

## Figures and Tables

**Table 1:** Survival rates (%) of honeybees across experimental groups over the 3 days.

Group	Survival (%) Day 1	Survival (%) Day 2	Survival (%) Day 3
CTRL	100	99.6	92.4
BPF	100	98	91.7
DMT 21.6	35.9	14.2	0
FLU 50	95.1	83.6	49.7
FLU 100	17.3	0	-
DMT 21.6 + FLU 50	89.5	77.6	75.2
DMT 21.6 + FLU 100	74.8	21.3	0
DMT 21.6 + FLU 50 + BPF	96.8	87.4	82.1
DMT 21.6 + FLU 100 + BPF	84.2	35.7	13.6

CTRL: control; BPF: bergamot polyphenolic fraction; DMT: deltamethrin; FLU: flupyradifurone.

## Data Availability

Data are available upon request to the corresponding author.

## Abbreviations

The following abbreviations are used in this manuscript:

BPF	Bergamot polyphenolic fraction
FLU	Flupyradifurone
DMT	Deltamethrin
